# The increasing importance of the oral microbiome in periodontal health and disease

**DOI:** 10.2144/fsoa-2023-0062

**Published:** 2023-06-12

**Authors:** Ruqaiyyah Siddiqui, Zahi Badran, Anania Boghossian, Ahmad M Alharbi, Hasan Alfahemi, Naveed Ahmed Khan

**Affiliations:** 1 College of Arts & Sciences, American University of Sharjah, Sharjah, 26666, United Arab Emirates; 2 Department of Medical Biology, Faculty of Medicine, Istinye University, Istanbul, 34010, Turkey; 3 Periodontology Unit, Department of Preventive & Restorative Dentistry, College of Dental Medicine, University of Sharjah, Sharjah, 27272, United Arab Emirates; 4 Department of Clinical Laboratory Sciences, College of Applied Medical Sciences, Taif University, Taif, 21944, Saudi Arabia; 5 Department of Medical Microbiology, Faculty of Medicine, Al-Baha University, Al-Baha, 65799, Saudi Arabia; 6 Department of Clinical Sciences, College of Medicine, University of Sharjah, University City, Sharjah, United Arab Emirates

**Keywords:** gingivitis, oral microbiome, oral microbiome; gut microbiome, periodontal pathogens, periodontitis

## Abstract

Herein, the aim is to discuss the current knowledge of microbiome and periodontal diseases. Current treatment strategies include mechanical therapy such as root planing, scaling, deep pocket debridement and antimicrobial chemotherapy as an adjuvant therapy. Among promising therapeutic strategies, dental probiotics and oral microbiome transplantation have gained attention, and may be used to treat bacterial imbalances by competing with pathogenic bacteria for nutrients and adhesion surfaces, as well as probiotics targeting the gut microbiome. Development of strategies to prevent and treat periodontal diseases are warranted as both are highly prevalent and can affect human health. Further studies are necessary to better comprehend the microbiome in order to develop innovative preventative measures as well as efficacious therapies against periodontal diseases.

## Plain language summary

This paper discusses what we currently know about the microbiome and periodontal diseases. Plaque buildup can happen for various reasons, like eating sugary foods, changes in saliva and the microorganisms in the mouth and gut. To treat periodontal diseases, we currently use antimicrobial medications and scaling. For patients with periodontitis, root planing and deep pocket debridement are used. Dental probiotics are getting attention as a potential treatment option. They work by competing with harmful bacteria for nutrients and surfaces in the mouth, and there are also probiotics that target the gut microbiome.

## Tweetable abstract

The microbiome plays a critical in periodontal health and disease, and thus should be targeted in dental therapies.

Periodontal diseases, which include gingivitis and periodontitis, are chronic inflammatory diseases, affecting the teeth supporting tissues. Their prevalence is estimated at 45–50% of individuals globally, with 11.2% of individuals diagnosed with severe periodontitis (Tables 1 & 2 [1–3]). Periodontal diseases are usually induced by dental plaque (microbial biofilm) and are associated with multiple species of bacteria that increase in numbers due to a dysbiosis of the plaque [4,5]. Chronic inflammation might promote the formation of periodontal pockets, which alter the redox and nutritional environment and boost the species richness and variety of biofilms leading to dysbiosis [6]. Gingivitis occurs due to the persistent accumulation of microbial biofilms, sometimes exacerbated by local risk factors such as calculus formation, teeth crowding, iatrogenic restoration, all of which are believed to be the main causes for the initiation and progression of gingivitis [7–13]. If left untreated, gingivitis may then progress into periodontitis: an inflammation characterized by alveolar bone loss, formation of periodontal pockets and ultimately tooth loss [7–13]. It has been observed that an alteration within the oral and gut microbiome occurs in periodontal diseases patients [14–16]. Additionally, changes in saliva composition have been observed in patients with changes in the saliva microbiota and amino acid concentrations [17,18]. Findings from a recent clinical study revealed that differences in salivary amino acid content in connection to clinical inflammatory symptoms may be influenced by periodontal condition and disease type. A strong association between methionine, citrulline, carnosine and arginine and clinical parameters, indicating that the amounts of various salivary free amino acids may be involved in periodontitis [18]. Herein, the role of the oral and gut microbiome in periodontitis and gingivitis are discussed. Using PubMed and Google Scholar databases, we examined the literature from the last decade, using key terms, namely “gut microbiome”, “oral microbiome”, “gingivitis”, “periodontitis”, “periodontal disease” and “microbial dysbiosis” with a focus on recent studies (2019–2022), and included all relevant and pertinent studies. Current treatment strategies include antimicrobial chemotherapy, root planing, scaling and deep pocket debridement. The development of novel treatment strategies such as the use of probiotics, oral microbiome transplantation and recent clinical studies are elaborated on.

**Table 1.** Incidence of gingivitis.

Incidence of gingivitis	Region/country	Age group
97% incidence rate	Iran	6–7 years
27% incidence rate	Yemen	5 years
78.6% incidence rate	Yemen	12 years
61.1% incidence rate	Greece	Athletes
60.6% incidence rate	Italy	Athletes
66.1% incidence rate	Spain	Athletes
78.5% incidence rate	Brazil	19 years
77.7% incidence rate	Kenya	12 years
52.6% incidence rate	Jordan	15 years
53.4% incidence rate	Ethiopia	12 years
47.6% incidence rate	United Arab Emirates	No specific age group
50–90% incidence rate	USA and UK	No specific age group

**Table 2.** Incidence of periodontitis.

Incidence of gingivitis	Region/country	Age group
47% incidence rate	USA	Adults over the age of 30 years
70% incidence rate	USA	65 years of age
20% of 164,036 subjects	United Arab Emirates	No specific age group
2.2% of 595 subjects	Jordan	No specific age group
8.6% of 2435 subjects	Saudi Arabia	High school students

## Clinical aspects & prevalence of gingivitis & periodontitis

**Figure 1.**
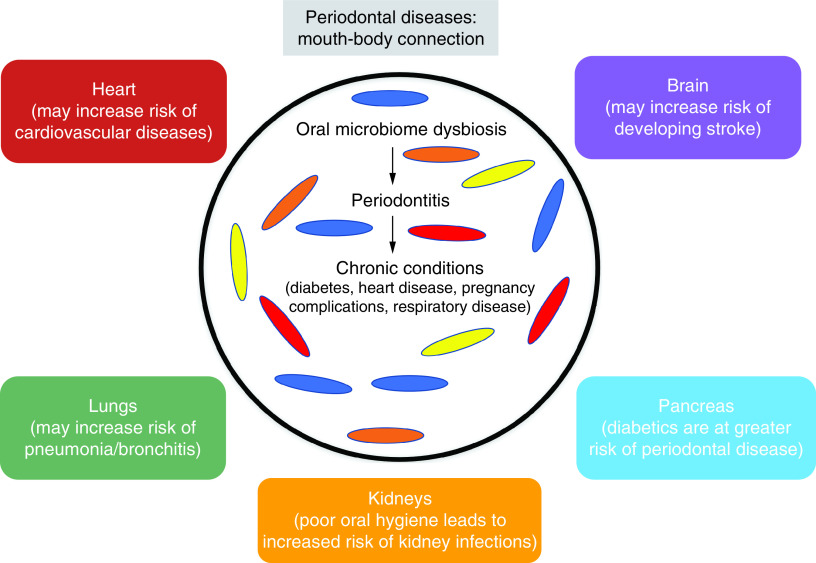
There is a strong association between periodontal disease and other chronic conditions such as diabetes, heart disease, pregnancy complications, respiratory disease, and so on.

According to the American academy of periodontology and the European Federation of Periodontology, gingivitis is a common oral infection, characterized by swelling, redness and inflammation of the soft tissues surrounding the teeth, the gingivae [7–9,19–22]. The clinical/diagnostic definition of dental plaque-induced gingival inflammation can be affected by various systemic and oral factors [4]. Furthermore, if left untreated, gingivitis may progress into periodontitis, a chronic oral inflammatory condition, initiated by the accumulation of pathogenic dental plaque biofilm above and below the gingival (gum) margin. Microbial dysbiosis may lead to a chronic nonresolving and destructive inflammatory response, characterized by the destruction of teeth supporting tissues, such as the alveolar bone and periodontal ligament and eventually tooth loss [7,8,20,23–27]. Additionally, periodontitis can also affect the overall health of an individual by increasing the risk of cancer, aspiration pneumonia, and atherosclerosis [28]. In a recent study, a prospective analysis to investigate the relationship between the severity of periodontal disease and the risk of cancer in older adults from black and white ethnic backgrounds was conducted. The study yielded further evidence suggesting that individuals with periodontitis have an increased risk of developing cancer, particularly lung and colorectal cancer. However, it is important to conduct additional research to gain a better understanding of the specific types of cancer affected and any potential variations based on race [29]. Periodontitis has been found to be associated with multiple diseases such as diabetes and cardiovascular disease (Figure 1) [30,31]. According to recent studies periodontitis was found to be potentially associated with age-related macular degeneration [4,30].

Fortunately, through early diagnosis, preventative measures against gingivitis can be taken [7,32,33]. An individual may be diagnosed with gingivitis if inadequate oral hygiene is maintained and/or immune system is impaired [7,23]. Additionally, one's diet and oral state plays a role in the occurrence of gingivitis [7]. Hence, through the maintenance of adequate oral hygiene such as: use of appropriate mouth rinses and brushing of teeth, an individual's chances of obtaining gingivitis can be reduced [21]. However, if periodontitis has occurred and has furthered into the stage of bone and tissue loss, then progression can only be slowed through plaque removal and reduction of inflammation [27].

## The oral microbiome connection in human health

A healthy oral microbiome is known to affect overall human health [8,34,35]. The term microbiome was first introduced by Joshua Lederberg, who defined it as an ecological community of symbiotic, pathogenic and commensal microorganisms; this refers to the microbes residing within our body [36–38]. The oral microbiome on the other hand refers to the microorganisms residing within the oral cavity [36,39]. Antony Van Leeuwenhoek, first identified the oral microbiome by observing his own dental plaque and reporting the movement of living animalcules [36,40–42]. Following the gut microbiome, the oral microbiome is the second largest and most diverse microbial community and most studied; and tends to exist in the form of a biofilm [26,36]. A microbiome consists of two parts: core and variable, the core being the common microbial to all individuals, whereas the variable being the microbiome influenced by lifestyle choices and physiology of an individual [36,41,43]. Bacteria in the oral cavity have two surfaces they can colonize: hard and soft tissues [36]. The tongue, cheeks, tonsils, hard palate, soft palate, gingival sulcus and teeth all provide rich environments on which microorganisms can grow and flourish, hence, the oral cavity is extremely diverse [33,36,37,40,44]. The surface of the oral cavity may contain a variety of bacteria leading to a bacterial biofilm [36].

Recently, the oral microbiome was studied within twenty healthy individuals through real-time quantitative PCR microarray and whole-genome sequencing (WGS). It was observed that each sample contained an oral environment constituting of various bacteria, fungi, protozoa and viruses. Results also revealed that within mucosal tissues the most represented genus of bacteria is Streptococci. Additionally, almost 700 species of prokaryotes, belonging to 185 genera and 12 phyla (*Fusobacteria*,* Firmicutes*,* Proteobacteria*,* Actinobacteria*,* Chlamydiae*,* Bacteroidetes*,* Spirochaetes*, SR1, *Synergistetes*,* Saccharibacteria* (TM7) and *Gracilibacteria* (GN02) within the oral microbiome were identified [36,41,45,46]. However, only 54% of species have been officially named, whereas 14% only have been cultivated and 32% are uncultivated phylotypes [36]. Additionally, protozoa, fungi and viruses were also found within the oral microbiome [36,40,45]. For example, *Trichomonas tenax* and the amoeba *Entamoeba gingivalis* are the two most common protozoa within the oral cavity [36,40,45]. The most common fungi associated with the oral cavity are *Candida*, although other species belong to *Cladosporium*,* Cryptococcus*,* Aspergillus*,* Saccharomycetales*,* Aureobasidium* and *Fusarium* have been found [36,40,45]. The viruses found within the oral microbiome are related to diseases and exhibit pathogenic characteristics, such as the herpes simplex virus 1 (HSV-1) or herpes simplex virus 2 (HSV-2) that causes *Herpes labialis*, commonly known to cause cold sores [40]. Hence, the oral microbiome is integral and maybe associated with multiple diseases such as diabetes, pancreatic cancer and podiatric Crohn's Disease, among others [8,34,35,46]. For example, studies have found that people with Type 2 diabetes have alterations in their oral microbiome compared with healthy individuals, including a decrease in beneficial bacteria and an increase in harmful bacteria. These imbalances may lead to inflammation, insulin resistance and other metabolic abnormalities that contribute to the development of diabetes [47]. One study found that individuals with pancreatic cancer had a distinct microbial signature in their oral microbiome compared with healthy controls, suggesting that these imbalances may play a role in the development of the disease [48]. Studies have found that imbalances in the oral microbiome may contribute to the development of Crohn's disease by triggering an inflammatory response in the gut [49]. Overall, the relationship between the oral microbiome and chronic conditions like diabetes, pancreatic cancer and Crohn's disease is complex and multifactorial. More research is needed to fully understand the mechanisms underlying these associations and to develop effective interventions to prevent and treat these conditions.

Recently, in a study conducted on Indian population, the oral microbiome was found to constitute 34% Proteobacteria, 32% Bacteroidetes, 24% Firmicutes, 6% Fusobacteria and 2% Actinobacteria [50]. In comparison, within the Emirati population the oral microbiome was found to be composed of Bacteroidetes, Actinobacteria, Proteobacteria, Firmicutes and Fusobacteria phyla, additionally *Neisseria*, *Haemophilus*,* Veillonella* and *Prevotella* genera were found. These results were similar to the oral microbiomes of Chinese, American, Japanese and Korean populations [46]. These studies indicate that differences in the composition of the microbiome across different human populations exist. This may be attributed to a combination of genetic, environmental and lifestyle factors, as well as cultural practices. Comprehension of the factors that contribute to microbiome variations will ultimately shed light on the relationship between the microbiome, health and diseases, and potentially guide personalized interventions and treatments in the future [51].

## Oral microbiome in gingivitis & periodontitis

The oral microbiome is the second largest microbiome and one of the most studied [36]. Oral microbial settlements are often found in either shedding or nonshedding surfaces; shedding surfaces include the cheeks, lips, palate, tongue and the gingival sulcus where epithelial cells are released from the epithelial junction, whereas nonshedding surfaces include the teeth, and artificial material such as orthodontic appliances, dentures, oral implants and tooth fillings [52]. It is on nonshedding surfaces such as teeth where dental plaque begins to form: these complexes of microbial ecosystems, also referred to as biofilms, may form on different locations, for example the fissure biofilm forms inside the teeth toward the dental pulp, whereas the supragingival biofilm forms on the dental enamel neighbouring the gingiva [39,45,52,53].

To keep a healthy oral ecosystem, equilibrium must be maintained, however, changes such as: puberty, poor oral hygiene, alcohol consumption, tobacco consumption, hormonal imbalance and stress may disrupt the oral equilibrium and lead to the development of various diseases including oral diseases [41]. Interactions among the oral microbiome, the host response and the development of periodontitis is a complex process. It is believed that the formation of a supragingival biofilm is related to gingivitis, as upon the biofilm's maturation and thickening, the facultative and anerobe species present cause gingival inflammation, ultimately causing gingivitis and periodontitis; this is often the result of an altered biofilm formation [38,52]. An altered biofilm is the result of equilibrium disturbances within the oral cavity microbiome, a condition known as dysbiosis, and may lead to periodontitis. Initially, the primary colonizers are Gram-positive aerobes and facultative anerobes like streptococci and *Actinomycetes* [45]. However, upon plaque maturation, the number of anerobic and Gram-negative bacteria increases, i.e., *Fusobacterium*,* Treponema* and members of *Synergistetes* were found to predominate [45,54]. This is likely due to disturbances within the microbiota and anerobic species tend to over dominate facultative species [38,52]. Furthermore, biofilm formation may cause the host to respond negatively by causing tissue damage through the initiation of uncontrolled levels of inflammation leading to an increased flow of nutrient-rich gingival crevicular fluid and bleeding, hence, the site of inflammation becomes oxygen deprived and anerobic microbes may dominate [52].

Since biofilm/dental plaque contribute to gingivitis and periodontitis, their removal is believed to slow the progression of the disease. A recent study revealed that upon tooth biofilm removal, *Firmicutes*, *Actinomyces* and *Veillonella* re-dominate initially, while gingivitis patient has *Actinomycetes*, *Campylobacter*,* Eiknella*,* Fusobacterium*, *Capnpcytophaga* and *Prevotella* dominating [39,53]. The anerobic species *Porphyromonas gingivalis*,* Treponema denticola* and *Tanerella forsythia* are believed to be associated with periodontal disease, species such as *Aggregatibacter actinomycetemcomitans* and *P. gingivalis* are found to survive and flourish [38,39,45,53,55]. In a study conducted in the UAE, *Fusobacteria* and *Firmicutes* were found in periodontitis patients [56]. Furthermore, *Synergistetes* species, *Dialister* species *Eubacterium saphenum*,* Anaeroglobus geminatus*, *Porphyromonas endodontalis*,* Filifactor alocis*,* Prevotella denticola*,* Parvimonas micra*, unnamed *Bacteroidetes* and *Fretibacterium* phylotypes were all determined to be disease associated [39,45]. It was found that upon mineralization of oral bacteria with calcium phosphate mineral salts, dental calculus can form [57]. The mineralized biofilms form subgingivally and supragingivally [57]. Furthermore, the nonmineralized dental biofilm were found to entrap oral bacteria, proteins and food remnants [57]. Moreover, strong associations have been shown between calculus deposits and periodontitis, and positive correlations have been made between the presence of calculus and prevalence of gingivitis [57]. Studying the oral microbial composition of healthy and infected individuals in different age and ethnic groups is necessary as this will allow a better understanding in the role of the microbiome and periodontal diseases.

## Gut microbiome connection in gingivitis & periodontitis

Oral bacteria have the capability of entering the circulation or transferring to the gut [15,16,58,59]. Periodontal pathogens like *T. denticola*,* A. actinomycetemcomitans* and *P. gingivalis* are able to invade endothelial cells [16]. Additionally, bacteria within the gut and oral cavity are found to overlap, in fact according to a study conducted 45% of bacteria found within the oral cavity is found in stool samples [15,16,58,59]. As explained earlier, periodontitis occurs as a result of an alteration in the oral microbiome, however, as certain oral bacteria transfer to the gut, the gut microbiome is disturbed and gut dysbiosis occurs as well [15,16].

For example, the bacterium *P. gingivalis* is a periodontal pathogen found in periodontitis patients [15,60]. This bacterium: *P. gingivalis* is swallowed in periodontitis patients and is transferred to the gut; although it is an oral bacterium, it is capable of surviving the acidic conditions of the stomach, hence, it is found to proliferate and reside within the gastrointestinal (GI) tract [15,16,61]. As a result, *P. gingivalis* leads to the alteration of the gut microbiome, leading to a gut dysbiosis [15]. The bacterium is also capable of resulting in metabolic disorders due to endotoxemia. In a study conducted, *P. gingivalis* was administered to mice, leading to a change in the gut microbiota compared with the sham-treated mice control [15,61]. The gut microbiota in *P. gingivalis* administered mice was found to be altered, with an increase in *Bacteroidetes* and decrease in *Firmicutes* [15]. Additionally, individuals with periodontal diseases such as periodontitis and gingivitis were found to obtain a less diverse gut/intestinal microbiome [15]. Moreover, *A. actinomycetemcomitans* is capable of altering the gut microbiota, as it is able to transfer to the gut as well. Furthermore, within the gut microbiome of individuals diagnosed with periodontitis, an increase in *Firmicutes*, *Euryarcheota, Proteobacteria* and *Verrucomicrobiota* was found; additionally, a decrease in *Bacteroidetes* was seen [15,16,60]. Moreover, the order *Lactobacillales* was found to be significant higher in gingivitis patients [16].

Given the limited available data, there is a need to analyse the oral microbiome shifts in periodontal disease patients under normal circumstances. Present studies focus mainly on oral microbiome shifts of smokers diagnosed with periodontal diseases. For example, in a study conducted in the UAE, subgingival plaque samples were collected from different types of tobacco smokers: shisha, medwakh and cigarettes. The samples were assessed for the different pathogenic makeup, and according to the results obtained *Veillonella dispa* and *Streptococcus mutans* was higher in cigarette smokers, *P. denticola* and *Treponema* sp. were higher in medwakh smokers, and finally *Tannerella forsythia* and *Streptococcus sanguinis* were higher in shisha smokers. The conduction of large-scale studies containing numerous subjects to study the oral microbial composition of healthy and infected individuals, will allow for a better understanding of the causative agents and consequences of periodontitis and gingivitis.

## Diet, saliva composition effects on microbiome & gingivitis/ periodontitis

Diet is known to affect both the oral and gut microbiome; and has been found to impact gingival/periodontal inflammation [62–64]. The consumption of high-glycaemic carbohydrates such as: white flour and sugar, as well as the consumption of saturated-, *trans*- and omega-6 fatty acids, while consuming low amounts of micronutrients and fibers promotes plaque accumulation and systemic inflammation, as well as impacting the microbiome [65]. Whereas, diet low in high-glycaemic carbohydrates, rich in omega-3 fatty acids (nonvegetable fats), vitamin C, vitamin D and fiber, have demonstrated reduction in gingival/periodontal inflammation, and the microbiome likely plays a role in mediating inflammation [62,63]. Such a diet has been shown to lead to an improvement in periodontal health [62,65].

The consumption of high-glycaemic carbohydrates and sugars has been shown to increase the risk of gingival bleeding, whereas a diet rich in complex carbohydrates such as: fruits, whole grain, vegetables and legumes resulted in the reduction of gingival inflammation and the incidence of gingivitis and periodontitis generally [62,63,65]. Recently, a correlation was found between the intake of sugar and gingival bleeding: an increase in gingival bleeding was associated with increased sugar intake [62,63,65]. One possible explanation is the promotion of microbial dysbiosis and chronic inflammation when carbohydrates are eaten excessively. It was shown that high glucose levels induce apoptosis and inhibit the proliferation of periodontal ligaments.

Moreover, the types of fat an individual consumes is associated with the overall health of an individual; with an imbalance between omega-3 and omega-6 fatty acids tend to result in inflammation [62,63,65]. Omega-6 fatty acids are considered pro-inflammatory fats and their consumption can result in changes in the microbiome [62,63,66]. Thus, the importance of diet and its effect on the microbiome, and resultant effects on oral health should be promoted. This is supported by a recent clinical study which examined the impact of an anti-inflammatory diet on various clinical, serological and subgingival microbiome measures [63,65]. For four weeks, the test group was required to switch to a diet low in animal proteins and processed carbs and high in omega-3 fatty acids, vitamin C, vitamin D, antioxidants, plant nitrates and fiber. Encouragingly, a noteworthy decrease in gingival inflammation was observed even in the absence of interdental hygiene [63,65].

The fluid covering the surfaces within the oral cavity or the saliva is a clear, slightly acidic, complex medium containing various enzymes and bacteria, and electrolytes such as calcium, sodium, potassium, phosphates, bicarbonate and magnesium [17,67]. Saliva maintains the oral homeostasis via the various proteins and enzymes it constitutes [17]. Salivary antimicrobials are important as they maintain a symbiotic relationship between host and resident microbiota. Additionally, saliva when secreted into the oral cavity is sterile, however, when sampled diverse microbiota is found. Of note, salivary microbiota of a healthy individual is different compared with a periodontitis patient. Studies conducted within Moroccan adolescents found that salivary levels of *A. actinomycemtomitans* were associated with clinical attachment losses [17], while in Japanese individuals, increased levels of *P. gingivalis* in the saliva was correlated with the percentage of sites with probing depths greater than or equal to 4 mm [17]. In Finnish individuals, *P. gingivalis, P. intermedia* and *T. forsythia* within the saliva were found to be associated with periodontitis [17]. Notably, probiotics have been found to influence the microbial community within the saliva. For example, the consumption of the probiotic strain *Lactobacillus casei* was found to significantly reduce the number of salivary *Streptococcus mutans* present [68].

## Current treatment strategies & prospective treatments using oral probiotics, oral microbiome transplantation & others

Gingivitis and periodontitis are the result of bacterial biofilm accumulation, hence, the removal of pathogenic bacteria from the oral cavity is essential, however this is a tedious task [3,69]. Currently various approaches are followed for the treatment of periodontal diseases including antimicrobials, root planing, scaling and, deep pocket debridement all of which attempt to reduce the number of pathogenic bacteria present [9,70–72]. These forms of treatments attempt to remove the pathogenic bacteria present because of bacterial biofilm alterations, which caused an imbalanced composition between pathogenic and commensal bacteria [9,73,74].

The most common nonsurgical form of treatment or periodontitis and often referred to as the gold standard is scaling and root planing [1,3,69]. This form of treatment effectively reduces microbial levels and reduces bleeding levels upon probing, with the aim of removing the subgingival biofilm, calculus and the bacterial toxins on the cementum surface [1,3,69]. The treatments are performed using a variety of dental tools such as hand instruments like curettes and periodontal scalers, sonic and ultrasonic instruments [3,69,75]. Although scaling and root planing are found to be relatively efficient, when manually done they are often time consuming and difficult; this is due to the unfavourable and complex root morphology when dentists are working blindly in deep pockets [69]. Additionally, it is not always able to eliminate all pathogenic bacteria in the subgingival plaque, that is because of their presence within deeper pockets to which the instruments may be harder to reach; hence, new instruments such as a power-driven ultrasonic mechanical instrument has been developed [69]. Unfortunately, simply removing the plaque may not be enough in all situations, as the bacteria have the ability to re-colonize after 8 weeks of treatment [69]. Hence, a combination of chemotherapeutic agents along with the scaling and root planing has been recommended, such as amoxicillin and metronidazole; yet, the use of systemic antibiotics is not preferred and is recommended for only refractory or rapidly progressing periodontitis [1,69,76]. Recently, a study investigated the efficiency of a phytotherapeutic medication made of plant extracts (composed of baicalin) on postoperative discomfort following molar surgery, in comparison to ibuprofen and a placebo, the phytotherapeutic medication made of herbal extract reduced the intensity of postoperative pain, likely affecting the oral microbiota [77]. Future studies to determine effects on gingivitis and/or periodontitis are warranted.

Chlorohexidine is one of the most effective local antimicrobials used in the treatment of periodontitis [78]. Chlorohexidine shows rapid antibacterial activity in periodontal pockets through the rapid attraction of the negatively charged cell surface of the bacteria to the cationic chlorohexidine molecule [78].

Another form of therapy for periodontitis is photodynamic therapy [79]. This form of therapy occurs in multiple ways, including the oxidation of cellular components like the DNA and plasma membrane, the photoactivation of exogenous or endogenous photosensitizers, and the formation of reactive species, all of which ultimately lead to cell death [79]. The process involves a photosensitizer reaction which results from a light source of a specific wavelength. The photosensitizer will then pass through various energy level states and finally attain an excite triplet state [79]. The state, results in the production of highly reactive singlet oxygen and toxic reactive oxygen species (ROS). These species are capable of penetrating cell membranes and inducing the oxidation of amino acids and membrane lipids [79].

To help treat bacterial imbalances, the use of probiotics is being explored. Probiotics are living nonpathogenic microorganisms believed to hold significant therapeutic potential upon administration in adequate amounts [70,73,74,80,81]. Probiotics were first introduced by Ilye Metchnikoff in 1908 [70,73,74,80,81]. Metchnikoff believed that due to greater consumption of lactic acid bacteria within fermented products, Bulgarians possessed an improved gastrointestinal health [70].

Probiotic strains can modulate inflammatory responses and produce lactic-acid, bacteriocin and hydrogen peroxide. Additionally, they can influence oxidative stress, inflammatory markers and systemic cytokines upon administration [9,70,74]. The following properties allow them to compete with pathogenic bacteria for adhesion surfaces and nutrients [9,70,74]. As a result, probiotic bacteria are found to better adhere to surfaces, resulting in the displacement of pathogenic bacteria. Furthermore, these properties may allow for a healthy biofilm formation [74]. In fact, in various studies conducted, the administration of probiotics lead to clinical improvement in periodontal patients in terms of bleeding upon probing [70,73,74,80,81].

The mechanism of action of probiotics in the oral cavity is not completely understood, but it is thought to involve a few different mechanisms. One proposed mechanism is that probiotics can compete with and displace pathogenic bacteria by occupying the same ecological niche in the oral cavity [26]. This may prevent the growth and colonization of harmful bacteria and thus reduce the risk of oral infections such as dental caries and periodontitis. Probiotics may also have immune-modulating effects, which can help to regulate the local immune response in the oral cavity. This may enhance the body's natural defense mechanisms against oral infections and promote tissue healing and repair [26]. Probiotics may also produce antimicrobial substances such as bacteriocins, which can directly inhibit the growth of pathogenic bacteria in the oral cavity [82]. Overall, the mechanism of action of probiotics in the oral cavity is likely to be multifactorial and may involve a combination of direct and indirect effects on the local microbial and immune environments [83]. However, further research is needed to fully understand the specific mechanisms by which probiotics exert their beneficial effects in the oral cavity.

*Lactobacilli* and *Bifidobacteria* are the most common strains of probiotics used [70,73,74,80,81]. Of most interest is the probiotic *Lactobacilli*, which has been found to inhibit *Prevotella nigrescens*, *Prevotella intermedia* and *P. gingivalis in vitro* [9,84]. Specifically, *Lactobacillus rhamnosus* is found to colonize and adhere to the oral cavity resulting in reduced periodontal pathogens [85]. In a previous study, it was found that the oral administration of *L. reuteri* led to 82% inhibition of *P. gingivalis* and 65% inhibition of *P. intermedia*; leading to reduced gum bleeding and swelling (gingivitis) [9,85].

In another study *Lactobacillus salivarius* WB21 containing soybean oil was used in chronic periodontitis patients, following a two-week treatment period a significant reduction in bleeding upon probing was found within the probiotic treated group [86]. Furthermore, reductions in *P. intermedia, T. forsythia, Fusobacterium nucleatum* and *P. gingivalis* was found [86]. Recently it is found that utilizing *L. salivarius* as a freeze-dried tablet, a reduction in *P. intermedia*, *P. gingivalis*, *T. forsythia*, *T. denticola* and *A. actinomycetemcomitans* was found [86,87].

Rather than using antibiotics to maintain a plaque-free environment, probiotics can be used. Probiotic strains such as *L. rhamnosus* and *Lactobacillus paracasei* are capable of binding to saliva-coated surfaces and competing with other bacteria. It is shown that mouthwash equipped with probiotics were given to study subjects and the effects of the mouthwash against periodontitis was studied [88]. A significant reduction in bleeding upon probing was found. Additionally, in the probiotic treatment group, probing pocket death was decreased, and a reduced gingiva inflammation was found. Similarly, probiotic chewing gums have been found to exhibit reductions in gingival inflammation [88,89]. In another study, a triphala mouthwash was developed; triphala is an ayruvedic medicine composed from the medicinal trees: *Emblica officinalis, Terminalia belerica* and *Terminalia chebula* [90]. Triphala has anti-inflammatory, antibacterial and antiseptic properties [90,91]. By modulating the human gut microbiota, the polyphenols in triphala may encourage the growth of advantageous *Bifidobacteria* and *Lactobacillus* while preventing the formation of unfavorable gut microbes. The gut bacteria stimulates the bioactivity of triphala to produce a range of anti-inflammatory chemicals [92]. Triphala mouthwash was found to reduce the number of oral streptococci in decayed and missing teeth. Additionally, significant decreases in plaque index and gingival index values were found in triphala treated groups compared with the placebo groups [90]. Furthermore, as nanotechnology is on a rise due to its effective treatment and better drug target-delivery properties, it can be used as novel antibacterial/antimicrobial agents [93]. More recently, the clay complex compound: cetylpyridinium chloride-montmorillonite was synthesized. Cetylpyridinium chloride is a cationic surfactant, whereas montmorillonite is a semectite clay. The compound when tested against bacteria residing within the human saliva, and was found to possess antibacterial properties at a concentration as low as 0.05 g/l.

Oral microbiome transplantation (OMT) is a promising new approach for the treatment of periodontal disease [94]. This involves the transfer of oral microbiota from a healthy individual to a patient with periodontal disease. The goal is to restore the balance of the oral microbiota, which is disrupted in periodontal disease and promote the growth of beneficial bacteria that can help to control inflammation and prevent further damage to the periodontal tissues. Several experiments have been conducted to evaluate the efficacy of OMT in the treatment of periodontal disease. One study found that OMT was effective in reducing inflammation and improving outcomes in dogs with periodontitis [95]. The data showed an “ecological shift” toward the composition of the donor microbiota, but this was transient in nature [95]. Clinical trials are needed to evaluate the safety and efficacy of OMT in the treatment of periodontal disease. These clinical trials will provide valuable information on the safety and efficacy of OMT, the optimal methods for performing the procedure, and any potential risks associated with the treatment. OMT is a promising new approach for the treatment of periodontal disease. While further research is needed to fully understand the safety and efficacy of OMT, the initial results are promising, and OMT may provide a new avenue for the treatment of this common and debilitating disease.

Recently *Akkermansia muciniphila* were administered in lean and obese mice serving as experimental periodontitis models. *A. muciniphila* is a mucophilic symbiont found within the colon of healthy individuals. It was found that upon the administration of *A. muciniphila*, alveolar bone destruction was reduced. Nevertheless, the common treatment options of scaling and root-planing may remove periodontal pathogens, but they do not prevent recolonization of pathogens; hence, the suggestion to utilize probiotics in combination with plaque removal. The use of lozenge containing *L. reuteri* was described recently as a lozenge once or twice a day for periodontitis patients in adjunct to scaling and root planning [9]. Chronic periodontitis patients assigned to use probiotic lozenges twice a day for a span of three months were found to possess significant probing pocket depth reductions and lower residual pocket depth size; additionally, reductions in the number of *P. gingivalis* were found [72,96]. Additionally, fermented milk containing *Lactobacillus curvatus* SMFM2016-NK was tested for its effect on periodontal disease in rats. It was found that the administration of *L. curvatus* SMFM2016-NK fermented milk alleviated periodontal inflammation and led to changes in the oral and gut microbiota. In order to evaluate the clinical and microbiological effects of locally administered *L. reuteri* probiotic as an adjuvant to scale and root planing in the treatment of chronic periodontitis, a study was carried out [97,98]. The outcomes demonstrated the probiotic *L. reuteri*‘s antibacterial effect as a promising supplementary therapy in improving periodontal parameters. To assess the full amount of the probiotic suspension's additional benefit, however, more extensive long-term trials with large sample sizes are required. Overall, the effect of probiotics in gingivitis and periodontitis are promising but remains species-dependent and results differ between studies conducted. Prospective clinical trials with potential probiotics and OMT are encouraged in order to suggest a probiotic strain, or combination of species, for the purposes of treatment, prevention/control and/or as an adjunct therapy to other treatment strategies. Recent technological developments in full automatic tooth segmentation methods using 3D cone-beam computed tomography could also explored in future studies [99].

## Conclusion

Based on the plethora of studies reviewed herein, it is evident that the microbiome plays an important role in the maintenance of optimal dental health, and thus should be targeted in dental therapies. Gingivitis and periodontitis are highly prevalent, impacting 45–50% of individuals globally [1–3]. Gingivitis is often associated with an imbalance in the oral and/or gut microbiome, changes in saliva composition and may progress into periodontitis. The removal of dental plaque is an important initial step to slow the progression of the disease; hence, current treatment strategies including root planing, scaling and deep pocket debridement to reduce numbers of pathogenic bacteria present, coupled with antimicrobial therapy. Novel therapies using stem cells, gene therapy, photodynamic therapy, 3D printing and layered biostructures for gingivitis and periodontitis are being considered [100]. Regeneration of periodontal tissues using gene therapy, cell-homing and layered materials are currently still in laboratory research phase [101,102]. Probiotics may be used to maintain a healthy oral microbiome, as certain probiotic strains may be able to compete with pathogenic bacteria for nutrients and adhesion surfaces. Oral microbiome transplantation may be able to alter oral microbiota and bring about a healthier state of ecological balances [103]. However, further studies regarding precise gut, oral and saliva compositions should be conducted. The collection of fecal and oral samples from patients and healthy individuals of various ages and ethnicities need to be conducted to allow for the identification of beneficial and detrimental microorganisms that may be present. In addition studies determining the effects of baicalin (phytotherapeutic drug) to the oral microbiome, as well as the use of artificial intelligence in dentistry [77,99]. This may ultimately lead to the identification of differences within the microbial composition in infected and healthy individuals, and further understanding within these complex interactions.

## Future perspective

Since the advent of next-generation sequencing and the launch of the human microbiome project, the oral microbiome has gained attention as a developing discipline. Symbiotic interactions occur in the oral microbiome, but when infections sever these bonds and lead to dysbiosis, a once-healthy environment can deteriorate and raise the risk of comorbidities. Additionally, it has been suggested that some microorganisms can operate as a bridge for other invasive infections, facilitating simpler migration. However, future studies in the next decade should focus on the mechanism by this occurs. Furthermore, more accurate models for periodontal disease pathogenesis are needed in order to better understand the role of bacteria in disease pathogenesis. It may be possible to pinpoint various taxa of bacteria within plaque biofilm to compare healthy and sick locations, as well as sites that deteriorate over time, with the use of future imaging tools to define the biogeography of plaque biofilm growth *in situ* during human disease. Additionally, a thorough examination of a greater variety of species, including previously uncultivated microbial species, in periodontal pathogenesis might shed light on their function in the pathogenesis of the disease. Finally, more clinical trials focused on targeting the microbiome and oral health are encouraged.

**Executive summary**

- In this article we discuss our current knowledge of microbiome and periodontal diseases.

**Clinical aspects & prevalence of gingivitis & periodontitis**

- Dental plaque-induced gingival inflammation is a common oral infection, that may progress into periodontitis, estimated to affect 45–50% of individuals globally.

**The oral microbiome connection in human health**

- An alteration in the oral microbiome of patients may occur due to periodontal pathogens such as *Porphyromonas gingivalis*.
- Microbial dysbiosis may lead to a chronic nonresolving and destructive inflammatory response, characterized by the destruction of teeth supporting tissues, such as the alveolar bone and periodontal ligament and eventually tooth loss.

**Gut microbiome connection in gingivitis & periodontitis**

- Build-up of plaque may be due to a plethora of factors, such as consumption of high-glycemic carbohydrates, saliva composition and changes in the oral and gut microbiome.
- These may promote plaque accumulation, systemic inflammation and result in imbalances in the oral and gut microbiome.

**Diet, saliva composition effects on microbiome & gingivitis/periodontitis**

- Diet is known to affect both the oral and gut microbiome; and has been found to impact gingival/periodontal inflammation.
- Of note, changes in saliva composition, in amino-acids and microbial composition has been observed in the saliva of gingivitis patients.

**Current treatment strategies & prospective treatments using oral probiotics, oral microbiome transplantation & others**

- Current treatment strategies for periodontal diseases include antimicrobials and scaling; whereas, root planting and deep pocket debridement are used specifically for periodontitis patients.
- Dental probiotics have recently gained attention and may be used to treat bacterial imbalances by competing with pathogenic bacteria for nutrients and adhesion surfaces, as well as probiotics targeting the gut microbiome.

**Concluding remarks & future perspectives**

- Further studies are necessary to better comprehend the oral, gut and saliva composition in order to develop innovative preventative measures as well as efficacious therapies against periodontal diseases.
- Accurate models for periodontal disease pathogenesis are needed in order to better understand the role of bacteria in disease pathogenesis, as well as for mechanistic studies.
